# Computational Fluid Dynamics in Predicting Renal Malperfusion After Aortic Dissection Repair

**DOI:** 10.1016/j.jaccas.2025.106060

**Published:** 2025-11-17

**Authors:** Chengwei Yang, Shujing Shao, Xiaochang Leng, Weiwei Qi, Ying Chen, Lianjun Huang, Lei Xu, Yuanming Luo

**Affiliations:** a Department of Imaging, Beijing Anzhen Hospital, Capital Medical University, Beijing, China; b Department of Imaging and Interventional Radiology, DeltaHealth Hospital Shanghai, Shanghai, China; c Department of Technology, Boea Wisdom (Hangzhou) Network Technology Co, Ltd, Hangzhou, China; d Department of Mechanical Engineering, The University of Iowa, Iowa City, Iowa, USA

**Keywords:** aortic dissection, computational fluid dynamics, renal malperfusion

## Abstract

**Background:**

Renal atrophy following aortic repair is difficult to be identified through renal function biochemical measures due to compensation. Computational fluid dynamics (CFD) analysis is a promising approach for identifying anomalies of renal blood supply, which is related to renal atrophy.

**Case Summary:**

This study reports 3 patients who underwent renal atrophy after aortic repair during follow-up. The hemodynamics of renal arteries in each case was analyzed through employing the CFD method. The pressure ratio (PR; renal artery-to-aorta) measurements revealed significantly lower values on the atrophic side of the renal artery when comparing to the nonatrophic side.

**Discussion:**

PR is promising to serve as an early quantitative indicator, with persistent depression prior to obvious morphological atrophy. Serial CFD-based PR assessments show promise in predicting the risk of renal atrophy and facilitating earlier intervention.

**Take-Home Message:**

Patient-specific CFD simulations based on computed tomography angiography offer hemodynamic profiling to detect perfusion compromise in the preclinical phases.

## Graphical Abstract

**Figure:**
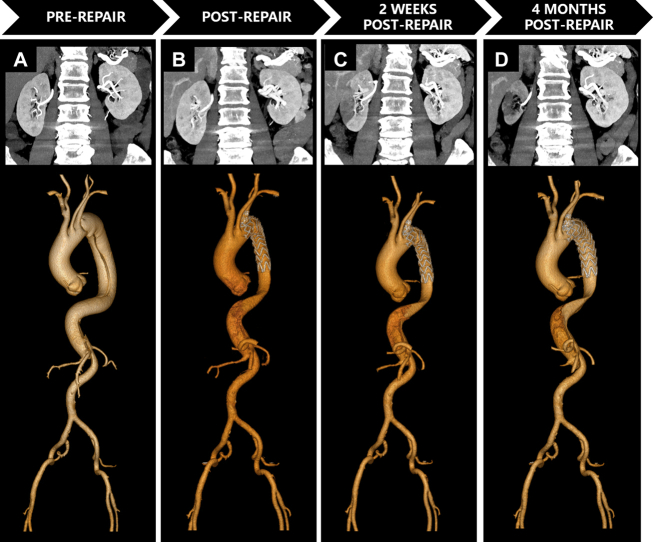

Renal artery malperfusion following aortic repair is a common complication that may lead to progressive atrophy. Early diagnosis and evaluation of malperfusion are critical for preventing irreversible kidney damage and malignant arterial disease. While there are ongoing advancements in this area,^1^^,^^2^ there is currently no established clinical diagnostic standard for malperfusion after aortic repair. Abnormal blood flow conditions in the aorta and its branches could present potentially an early indication of malperfusion.^3^ Hemodynamic analysis may be able to identify such alterations early on. Nevertheless, it is currently unclear how hemodynamics and perfusion of renal arteries are related.

**Take-Home Message**

• Patient-specific computational fluid dynamics simulations based on computed tomography angiography offer hemodynamic profiling to detect perfusion compromise in the preclinical phases.

The hemodynamic analysis based on computational fluid dynamics (CFD) offers a completely new way to investigate malperfusion of the renal artery. By reconstructing patient-specific vascular models, CFD simulates the blood flow inside blood arteries. Due to the complicated pathophysiology of renal artery malperfusion following repair, there are currently no established quantitative metrics for assessing renal artery perfusion, which restricts the use of the CFD approach in clinical settings.

This study employed CFD for patient-specific hemodynamic simulation of 3 patients with malperfusion after aortic repair. Analyze the causes of poor renal artery perfusion following aortic repair and try to find a quantitative parameter to assess the renal artery's perfusion condition.

## Data and Methods

### Patient 1

A 71-year-old male was transferred for management of Stanford type B aortic dissection (AD). Thirteen days prior to admission, he developed sudden retrosternal oppressive pain and profuse diaphoresis triggered by alcohol consumption. Symptoms resolved spontaneously after rest. Initial evaluation at an external hospital confirmed Stanford type B AD via computed tomography angiography (CTA). He was subsequently transferred to our institution for definitive treatment.

The patient underwent elective thoracic endovascular aortic repair under general anesthesia. A single-branched Castor aortic stent-graft (36-30-200 mm; MicroPort Medical) was implanted, where the proximal anchoring zone was located at the aortic arch, achieving left subclavian artery preservation. The contrast agent administered to patient 1 during the procedure was Iopamidol (300 mgI/mL; Bracco Imaging), with a total dosage of 80 mL. The patient was discharged on postoperative day 4 without complications.

### Patient 2

A 39-year-old female with chronic Stanford type B AD was urgently admitted for acute exacerbation of tearing chest/back pain with diaphoresis persisting for 2 hours. Her dissection history dates to one and a half years ago when diagnosed via CTA at a regional hospital and managed medically with strict blood pressure control. Three months ago, surveillance CTA at another hospital revealed DeBakey type III dissection progression with pericardial effusion and left-lower-lobe subsegmental atelectasis.

The patient underwent total aortic arch replacement with frozen elephant trunk implantation combined with left common carotid-to-subclavian artery bypass under general anesthesia. During the surgery, a stent-graft with a diameter of 24 mm and a length of 120 mm was used for the elephant trunk segment (MicroPort Medical). Technical success was confirmed by complete false lumen exclusion on intraoperative imaging.

The patient achieved uncomplicated recovery and was discharged on postoperative day 7. The postoperative course was uneventful with patent bypass graft confirmed by Doppler ultrasound.

### Patient 3

A 54-year-old male developed sudden, severe tearing chest/back pain with diaphoresis without prodrome. The initial CTA at a regional hospital confirmed acute Stanford type A AD. Despite medical management (blood pressure control, analgesia, oxygen therapy), surveillance CTA revealed disease progression in 2 days: new moderate pericardial effusion and bilateral pleural effusions.

The patient underwent urgent surgical repair on the day of admission, consisting of 1) ascending aorta replacement with a 24-mm tetrafurcated graft (Terumo); 2) total arch reconstruction with a 22 × 100-mm frozen elephant trunk implantation (MicroPort Medical); and aortic valve repair. The duration of intraoperative cardiopulmonary bypass was 123 minutes, and the duration of circulatory arrest was 12 minutes. Postoperative hemodynamics remained stable, with no neurological deficits observed during intensive care unit monitoring.

More clinical information and past medical history are summarized in Table 1.

**Table 1:** Clinical Information of the Patients

Clinical	Patient 1	Patient 2	Patient 3
Age, y	71	39	54
Sex	Male	Female	Male
AD type	Stanford B	Stanford B	Stanford A
Diabetes	No	No	No
Hypertension	No	Yes	No
CKD (before AD)	No	No	No
COPD	No	No	No
History of surgery	lumbar spine surgery	No	No
Renal function tests (postoperation)			
BUN (mmol/L)	6.6	3.5	17.3
SCR (μmol/L)	76	76	154
GFR (mL/min/1.73 m^2^)	84	78.3	43.4
Renal function tests (follow-up endpoints)			
BUN (mmol/L)	6.4	3.7	8.5
SCR (μmol/L)	114	79	96
GFR (mL/min/1.73 m^2^)	55.4	80.1	76.9

AD = aortic dissection; BUN = blood urea nitrogen; CKD = chronic kidney disease; COPD = chronic obstructive pulmonary disease; GFR = glomerular filtration rate; SCR = serum creatinine.

### Aortic CT image acquisition

**Figure 1:**
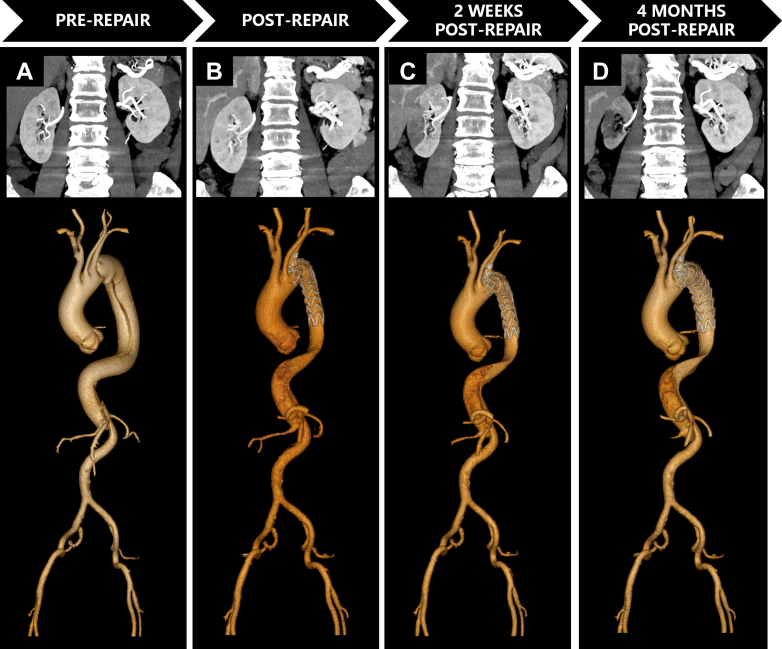
Serial Computed Tomography Angiography Imaging Demonstrating Aortic Evolution and Progressive Renal Atrophy in Patient 1 (A) Preoperative CTA: Stanford type B AD involved right renal artery (RRA) origin; left renal artery (LRA) originates from true lumen (TL) with preserved bilateral cortical enhancement and normal corticomedullary differentiation. (B) Immediate post-repair CTA: Patent RRA with adequate contrast opacification and maintained renal volumes. (C) Two-week CTA: Persistent RRA opacification but new segmental cortical hypoperfusion with ostial stenosis. The false lumen (FL) volume remains stable. (D) Four-month CTA: Critical RRA stenosis, significant renal volume loss (73% of preoperative), and near-absent parenchymal perfusion—classic.

**Figure 2:**
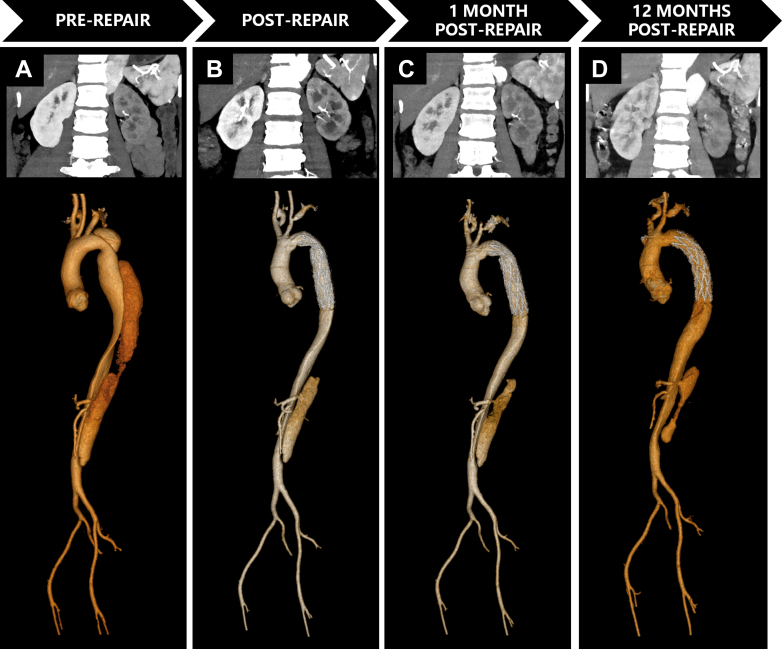
Serial Computed Tomography Angiography Demonstrating Aortic Evolution and Progressive Renal Atrophy in Patient 2 (A) Preoperative: Stanford type B AD involved renal arteries. LRA originates from FL, RRA from TL with left renal hypoperfusion despite symmetric volumes. (B) Immediately postsurgery: Persistent hypoperfusion of the left kidney. (C) One-Month CTA: FL thrombosis with mild volume reduction. Left renal hypoperfusion persists, but volume remains preserved. (D) Twelve-Month CTA: Complete FL thrombosis extending to LRA origin causing critical inflow obstruction. The left kidney volume lost (18% preoperative volume) and cortical thinning. The right kidney showed compensatory hypertrophy.

**Figure 3:**
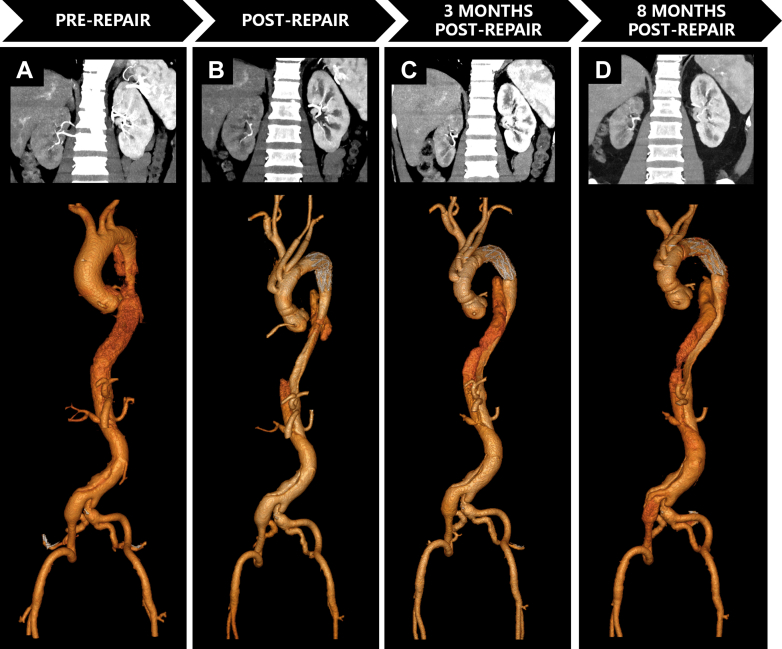
Serial Computed Tomography Angiography Demonstrating Aortic Evolution and Progressive Renal Atrophy in Patient 3 (A) Preoperative CTA: Stanford type A AD involved visceral segment. The RRA arises from the FL, LRA from the TL. Right renal hypoperfusion with preserved bilateral volumes. (B) Postoperative CTA: Midaortic FL thrombosis. Persistent right renal hypoperfusion. Volumes symmetric. (C) 3-Month CTA: Retrograde FL recanalization bridging suprarenal and infrarenal segments. Exacerbated right hypoperfusion with >25% volume loss. (D) 8-Month CTA: Persistent FL patency, progressive right atrophy (47% preoperative volume). Left kidney compensatory hypertrophy.

Aortic CTA was performed on a 128-slice CT scanner (Ingenuity CT, Philips Healthcare). Iomeprol (400 mgI/mL; Bracco Imaging) was administered intravenously at 3.5-4.5 mL/s (total volume 65-75 mL) (Figures 1, 2, and 3).

### CFD analysis of aortic hemodynamics

**Figure 4:**
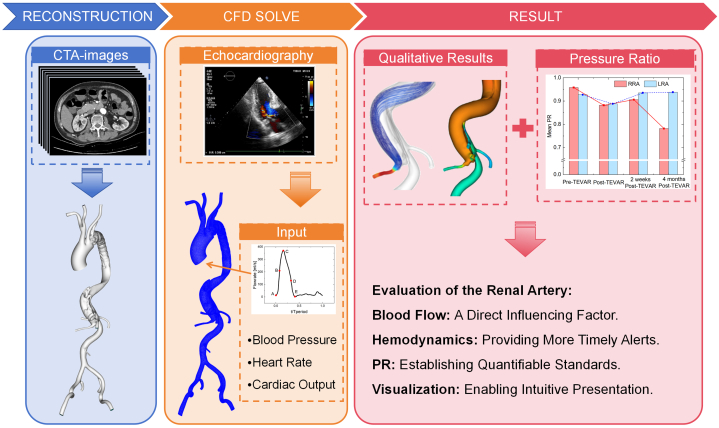
Workflow for Patient-Specific Computational Fluid Dynamics Assessment of Renal Artery Hemodynamics

CTA images were used to create patient-specific 3D vascular models using DetecModeling software (v3.2; Boea Wisdom Technology).^4^ CFD simulations were then performed using DetecFluid software (Boea Wisdom Technology).^5^ The patient-specific input flow curve was created by combining echocardiography-derived physiological characteristics and a standardized ascending aortic flow waveform, which served as computational boundary condition for hemodynamic simulations. Figure 4 provides a detailed illustration of the CFD procedure.

### Hemodynamic parameters

Pressure and velocity distributions inside the vascular model serve as the foundation for quantitative hemodynamic analysis. This study defines the pressure ratio (PR) as a hemodynamic measure that quantifies the proportionate relationship between renal artery pressure and aortic pressure:

$$\mathrm{PR}=\frac{P_{r}}{P_{a}}$$

where $P_{r}$ is renal artery pressure, and $P_{a}$ is aortic pressure.

## Results

This study compared flow velocity, pressure, and PR values of the renal artery region in 3 patients before surgery, after surgery, and during follow-up period. Assess the predictive value of the CFD approach in identifying renal artery malperfusion following aortic repair by comparing the PR of the atrophic side to that of the nonatrophic side.

### Patient 1

**Figure 5:**
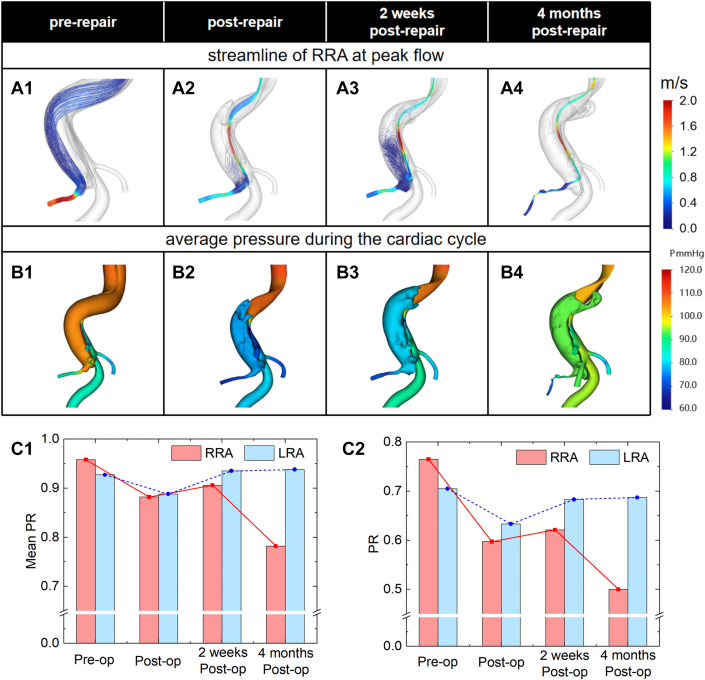
Computational Fluid Dynamics Simulation Results of Patient 1 (A) Streamlines at the peak of blood flow, (B) average pressure during the cardiac cycle, (C1) average PR value of renal arteries during the cardiac cycle, and (C2) PR value of renal arteries at the peak of blood flow.

Preoperatively, the RRA was perfused from the FL via the proximal entry tear. Postoperative tear repair shifted flow to a distal re-entry tear, inducing distinct vortices within the FL and at the RRA ostium (Figure 5a2 and a3). Concurrently, the pressure of FL decreased compared to preoperative level. By 2 weeks, persistent FL hypotension created a localized low-pressure zone at the RRA ostium. At 4 months, mild FL pressure recovery failed to reverse the RRA's irreversibly depressed pressure.

Compared to preoperative baselines, the average PR showed an 8% decrease in RRA against 4% in LRA. Peak-flow analysis revealed more pronounced differences: instantaneous PR of RRA decreased 22% postoperatively compared to LRA's 10% reduction. By 2 weeks, the PR of LRA had returned to baseline, but the PR of RRA remained much lower than the preoperative level.

### Patient 2

**Figure 6:**
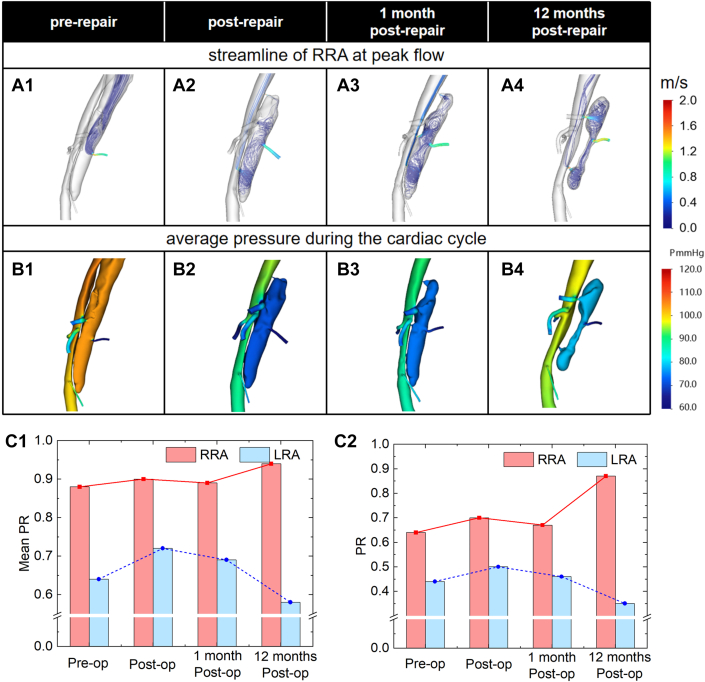
Computational Fluid Dynamics Simulation Results of Patient 2 (A) Streamlines at the peak of blood flow, (B) average pressure during the cardiac cycle, (C1) average PR value of renal arteries during the cardiac cycle, and (C2) PR value of renal arteries at the peak of blood flow.

As shows in Figure 6, Antegrade flow from the proximal FL entry tear generates low-velocity flow to the LRA before the surgery, but the pressure gradient between the FL and LRA was significant. The LRA's postrepair flow altered trajectory due to a proximal re-entry tear. After 1 month, chaotic turbulent flow was generated by partial FL thrombosis near the aortic renal portion. At 12 months, there was a noticeable decrease in velocity and pressure as the FL thrombus entered the LRA ostium.

The average PR of LRA was 26% lower than that of RRA before surgery. Even while postoperative intervention increased the average PR of LRA recovery to 0.72, it was still significantly less than RRA. At 1 month, the average PR of LRA had dropped by 12% from postoperative values, and by 12 months, the total had dropped by 20%.

### Patient 3

**Figure 7:**
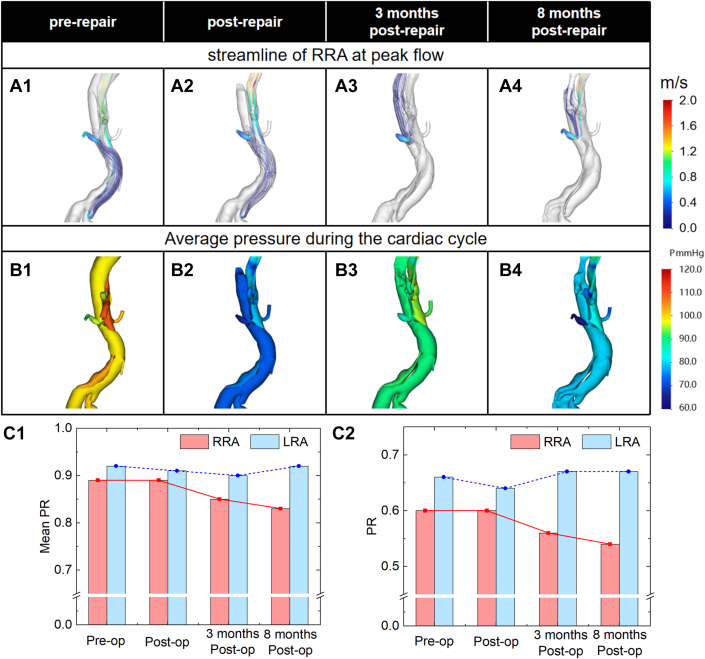
Computational Fluid Dynamics Simulation Results of Patient 3 (A) Streamlines at the peak of blood flow, (B) average pressure during the cardiac cycle, (C1) average PR value of renal arteries during the cardiac cycle, and (C2) PR value of renal arteries at the peak of blood flow.

Preoperatively, FL supplied LRA via retrograde perfusion from iliac inflow, maintaining near-equilibrium pressure between lumina. Postoperatively, FL volume decreased, yet iliac inflow persisted as the primary LRA source. At 3 months, LRA flow returned to antegrade perfusion as FL expansion restored continuity through a proximal entrance tear. These changes can be clearly seen in Figure 7.

Preoperatively, both renal arteries had comparable average PR values. After surgery, there was no evident acute PR decline in either artery. After 3 months, the RRA showed a noticeable PR decrease. This decrease accelerated at 8 months, leading to persistent depression. The PR of LRA indicated a minor PR increase during follow-up.

## Discussion

### CFD analysis of renal perfusion

Early detection of branch artery malperfusion and intervening at optimal timing after aortic repair remains a great clinical challenge. Kidney is an important organ depending only on single-artery perfusion. Once the renal artery presents perfusion abnormality, ischemic injury would progress in the associated kidney.^6^ Although the serum creatinine generally serves as a biomarker for renal injury, its sensitivity is not better than 70% due to the strong compensatory mechanisms of the human body.^7^ CFD demonstrates utility in visualizing hemodynamics of arteries, but prior renal atrophy studies focused primarily on correlating atrophy with hemodynamic patterns, flow velocity, pressure, and wall shear stress.^8^^,^^9^ A quantification indicator for the evaluation of atrophy severity is still lacking, which limits the application of CFD analysis on this focus.

This report presents 3 cases of renal artery atrophy pathogenesis following AD repair, each demonstrating distinct hemodynamic characteristics. In case 1, surgical intervention within the FL induced complex vortex formation at the renal artery ostium. Although the overall FL volume remained stable during follow-up, this localized flow disturbance significantly impaired perfusion to the FL-dependent renal artery. In case 2, progressive thrombosis of the FL during follow-up led to thrombotic extension into the renal artery, resulting in critical perfusion reduction. In case 3, the FL experienced complex thrombosis followed by partial recanalization. Although no pathological vortices were detected, severe stenosis developed at origin of the FL-supplied renal artery, leading to substantial hemodynamic compromise. Based on these observations, this study proposes a quantitative metric to evaluate the severity of renal artery atrophy arising from different underlying hemodynamical mechanisms.

### PR in clinical decision-making

Recently, fractional flow reserve (FFR), as a pressure-derived index, has been widely adopted for coronary artery hemodynamics assessment in clinics. This successful clinical translation underscores the value of pressure gradient alterations across vascular segments as quantifiable indicators of regional perfusion status. Similarly, the application of renal fractional flow reserve (rFFR) for evaluating renal artery stenosis has attracted growing attention.^10^ In contrast, CFD-derived PR offers notable advantages including noninvasiveness and cost-effectiveness.

Both transient and mean PR exhibited postoperative fluctuations, which manifests as variable increases or decreases due to surgically induced hemodynamic patterns in TL and FL. During subsequent follow-ups, the PR of the affected renal artery consistently declined across all cases, whereas the contralateral renal artery maintained a comparable PR value to preoperative baselines. Notably, this decline in PR consistently preceded detectable impairment in renal function. These disparities were more pronounced at peak systolic flow. While minor variations in boundary conditions may influence absolute PR values, this study emphasizes comparative trends between bilateral renal arteries to mitigate such effects.

The most important function of this method is to identify the risk of renal artery atrophy in the early postoperative period and enable timely intervention. When such risks are detected, the timing of intervention can be assessed by integrating clinical judgment with hemodynamic findings. In addition, the location and size of the tear near the renal artery should be evaluated, followed by intervention measures such as balloon dilatation of the tear at the renal artery level or endovascular stenting of the renal artery.

From these 3 cases, regardless of the FL's progression pattern or the specific cause of renal artery hypoperfusion, PR consistently exhibited the same trend and demonstrated predictive value. Crucially, the decline of PR consistently preceded radiographically confirmed renal atrophy, regardless of the morphology of FL. This uniform pattern establishes PR as an etiology-agnostic early biomarker, transcending conventional anatomical classifications and offering a means of risk stratification before irreversible parenchymal loss occurs.

### Clinical implications and study limitations

In this report, flow pattern analysis successfully predicted branch artery perfusion status during FL remodeling. Both PR and velocity measurements prove effective in assessing renal perfusion, with sustained postoperative PR reductions beyond 2 weeks emerging as potential early indicators of stenosis. While this study introduces a novel approach for predicting renal malperfusion in AD, its single-sample design necessitates validation in larger patient cohorts. Future studies should aim to expand the sample size and establish standardized thresholds for early PR decline and flow reduction, thereby guiding timely clinical intervention.

## Funding Support and Author Disclosures

This research was partially supported by Shanghai Qingpu District Special Discipline Support Project (No. TZ2023-13). The authors have reported that they have no relationships relevant to the contents of this paper to disclose.
